# New integrated three-interface conceptual model for clearance and immune surveillance in the human CNS

**DOI:** 10.3389/fcell.2026.1785072

**Published:** 2026-04-16

**Authors:** José García-Cosamalón, Miguel Marchena, Vega Villar-Suárez, Luis Ley, Fernando de Castro

**Affiliations:** 1 Instituto de Biomedicina, Universidad de León, León, Spain; 2 Centro de Neurociencias Cajal/Instituto Cajal, Consejo Superior de Investigaciones Científicas-CSIC, Madrid, Spain; 3 Faculty of Health Sciences – HM Hospitals, Universidad Camilo José Cela-UCJC, Madrid, Spain; 4 HM Hospitals Health Research Institute, Madrid, Spain; 5 Servicio de Neurocirugía, Hospital Universitario Ramón y Cajal-SERMAS, Madrid, Spain

**Keywords:** AQ4, arachnoid granulations, astrocyte, brain clearance, cerebrospinal fluid, endfeet, glia, glymphatic segmentation

## Abstract

The central nervous system (CNS) lacks a classical lymphatic system and instead relies on cerebrospinal fluid (CSF) circulation for metabolic waste removal and for defense/immune surveillance. Recent research has consolidated the concept of an integrated glymphatic–lymphatic continuum (because of the relevant contribution of glial cells), where CSF acts as a central transport medium linking parenchymal exchange with meningeal lymphatic outflow. Within this framework, the glymphatic system supports CSF–interstitial fluid exchange, particularly during sleep, when vascular pulsatility enhances solute clearance. Translating insights from rodent systems to the human brain, however, requires explicit consideration of the subarachnoid space and CSF hydrodynamics as an intermediate interface that distributes CSF and modulates the balance between parenchymal influx and efflux toward dural lymphatics. We here propose a three-phase conceptual model comprising: (1) a microscale segment that includes perivascular spaces, the neuropil/interstitial compartment, glial elements, and aquaporin-mediated water transport; (2) a mesoscale segment centered on the arachnoid barrier and the subarachnoid CSF compartment, functioning as a distribution and exchange interface; and (3) a macroscale segment encompassing arachnoid granulations and dural meningeal lymphatic vessels as the principal egress pathways. To further support this with a clinical perspective, we review evolutionary adaptations that have resulted in more efficient fluid exchange between CSF reservoirs and the extracellular space of the human brain. This three-interface framework may improve diagnostic and therapeutic precision by recognizing that the distinct disorders, such as neurodegenerative proteinopathies, cerebral edema, hydrocephalus, or meningoencephalitis, preferentially disrupt specific interfaces across scales.

## Introduction

In the absence of conventional lymphatic vessels within the brain parenchyma, evolutionary forces appear to have driven the emergence of a specialized system for waste clearance: the glymphatic system or glymphatics (Iliff et al., 2012). Over the past decade, the “glymphatic system” has reshaped our understanding of cerebrospinal fluid (CSF), highlighting its active role in maintaining brain homeostasis rather than serving merely as a passive cushioning medium. Mostly derived from rodent tracer experiments, its original formulation proposed that CSF enters the brain through periarterial spaces, exchanges with interstitial fluid (ISF) through astroglial endfeet enriched in aquaporin-4 (AQP4), and exits along perivenous routes, thereby facilitating the clearance of metabolites and macromolecules from neural tissue (Iliff et al., 2012; Hablitz and Nedergaard, 2021). This influential paradigm provides a mechanistic link between fluid dynamics, sleep–wake state, vascular pulsatility, and the removal of neurotoxic proteins (e.g., amyloid-β and tau) involved in neurodegeneration (Iliff et al., 2012; Xie et al., 2013; Mestre et al., 2018a). In parallel, the rediscovery of meningeal lymphatic vessels in the dura mater provided an anatomical “outflow” endpoint supporting a simplified two-interface narrative: an intraparenchymal glymphatic influx/efflux interface directly coupled to dural lymphatic drainage (Aspelund et al., 2015; Louveau et al., 2015). This glymphatic–meningeal lymphatic axis is conceptually attractive and experimentally affordable in rodents with small brains, high surface-to-volume ratio, and relatively direct ventral routes between CSF compartments and cranial lymphatics that facilitate tracer-based mapping (Kida et al., 1993; Ma et al., 2017; Brady et al., 2020).

However, such a two-interface framing risks underrepresenting the importance of the subarachnoid space (SAS) and CSF itself (the carrier phase for glymphatic exchange) because the SAS constitutes the major extracerebral space through which solutes transit before reaching final efflux pathways (Matsumae et al., 2016; Plá et al., 2023). New structural data argue that the SAS is not a uniform but a compartmentalized mixing chamber; a biologically active interface (Yasargil, 1984; Johanson et al., 2008). A thin mesothelium-like layer (the subarachnoid lymphatic-like membrane (SLYM)) divides the SAS into distinct inner and outer compartments, providing a plausible anatomical substrate for size-selective filtration and immune surveillance within CSF channels (Møllgård et al., 2023). Therefore, “subarachnoid processing” of CSF-borne solutes may be spatially organized and regulated, rather than merely downstream of glymphatic outflow (Møllgård et al., 2023; Plá et al., 2023). Recently, arachnoid granulations (AGs), classically considered as simple one-way pressure valves into venous sinuses in humans, have been proposed as lymphatic-associated conduits that communicate with dura-arachnoid-stroma and even adjacent marrow spaces: therefore, AGs may be exit SAS gateways for the CSF into dural/interstitial compartments that interface with immune and lymphatic networks (Shah et al., 2023). Complementing these, human perivascular pathways show functional heterogeneity and disease-relevant alterations, further underscoring the need to integrate microanatomy with extracerebral CSF pathways rather than considering the latter as a homogeneous sink (Weller, 1998; Tarasoff-Conway et al., 2015; Eide and Ringstad, 2024).

On this basis, we propose here that the prevailing rodent “direct glymphatic–lymphatic coupling” model is incomplete for capturing the human clearance continuum, which should explicitly incorporate the SAS/CSF as an intermediate, structured interface between intraparenchymal exchange and final lymphatic elimination. Clearance and immune-vigilance are organized across three interdependent scales: (i) a microsegment (intraparenchymal perivascular CSF–interstitial fluid exchange, astroglial–vascular coupling, and local neuroimmune regulation), (ii) a mesosegment (subarachnoid compartmentalization, cisternal/perivascular geometry, and CSF-borne mixing with SLYM-associated filtration and immune surveillance), and (iii) a macrosegment (arachnoid granulation-mediated CSF egress, dural venous/perineural outflow routes, and meningeal lymphatic drainage; Table 1). This segmentation is intended to reconcile rodent mechanistic insights with emerging human anatomy, while providing a modular scaffold to interpret site-specific vulnerabilities, whether they are primarily intraparenchymal or extra-axial (subdivided into subarachnoid and dural/lymphatic).

**TABLE 1:** Three-interface model of CNS clearance.

Micro-scale	Meso-scale	Macro-scale
Intra-axial (parenchymal)	Extra-axial/subarachnoid	Extra-axial (dural)
Perivascular space/glia AQP4–ISF–neuropilIPAD	Arachnoid -CSF interface Piamater: subpial space SLYM	Dural meningeal lymphatics Arachnoid granulations venous sinuses-CribR
Glymphatic	Lymphatic-like	Lymphatic

AQP4, aquaporin-4; ISF, interstitial fluid; IPAD, intramural periarterial drainage; SLYM, subarachnoid lymphatic-like; CribR, cribiform (olfactory/nasal) route.

## Perivascular spaces and CSF–ISF exchange: the microsegment

**FIGURE 1:**
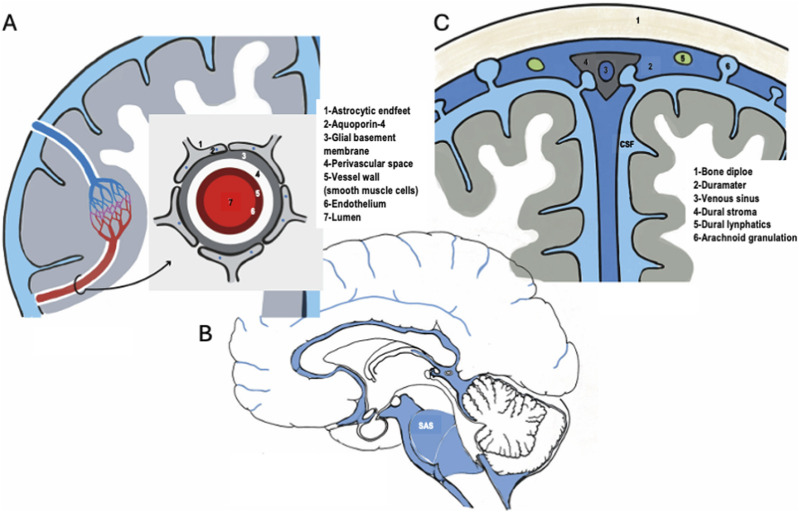
New integrated three-interface conceptual model for clearance and immune surveillance in the human CNS. **(A)** The microscale segment shows arterial and venous vessels, along with the relevant contributions of astrocytes with their AQ4-rich endfeet. The inset shows a transverse view of the organization of the vessels, the perivascular space, and the astrocytic endfeet. **(B)** Sagittal section of the human brain showing the mesosegment interface (in blue), including the subarachnoid space (SAS). **(C)** Schematic representation of the macrosegment, including the dural structures (dura mater itself, the dural venous sinus, the dural stroma, dural lymphatics, and the arachnoid granulations (AG).

The perivascular space (PVS) is the principal micro-interface coupling cerebrospinal CSF dynamics to interstitial fluid (ISF) transport within the brain (Bohr et al., 2022). In rodents, CSF enters the CNS along periarterial PVS surrounding penetrating arteries, distributes into parenchymal interstitium and neuropil, and exits along perivenous routes, generating a directional CSF–ISF exchange pathway that accelerates the clearance of solutes, including amyloid-β (Iliff et al., 2012). This “glymphatic” framework highlights the anatomical continuity between vascular perivascular spaces and astrocytic endfeet covering microvasculature, suggesting that neurovascular unit geometry is a key determinant of brain-wide macromolecular transport (Iliff et al., 2012; Mestre et al., 2018b; Troili et al., 2020; Figure 1A). A subpopulation of oligodendrocyte precursor cells has been identified as a contributor to the integrity of the blood–brain barrier in young adult rodents (Maki, 2017; Pfeiffer, 2022).

Mechanistically, CSF–ISF exchange seems regulated by vascular pulsatility and state-dependent changes in extracellular volume fraction. Reduction of arterial pulsatility and particle-tracking in experimental hypertension decreases periarterial influx and slows CSF–ISF exchange *in vivo*, supporting a role for the cardiac cycle as a driver of perivascular transport (Iliff et al., 2013; Mestre et al., 2018b). In contrast, sleep and several anesthetic regimens increase the interstitial space volume fraction and enhance solute clearance, suggesting that CSF–ISF transport strongly depends on tissue hydraulic properties and arousal state (Xie et al., 2013). Aquaporin-4 (AQP4), highly expressed in astrocytic endfeet, regulates water permeability and the exchange between CSF and ISF. It is essential for the clearance of cerebral metabolic waste through the glymphatic system, whose experimental dysfunction is associated with reduced clearance of injury-related solutes (for example, tau in traumatic brain injury models) and neurodegenerative alterations (Iliff et al., 2014; Salman et al., 2022). However, the relative importance of bulk flow, dispersion, and diffusion in solute transport remains debated, as tracer movement does not necessarily indicate a sustained convective flux through the interstitium. Evidence suggests that multiple transport regimes coexist (Johanson et al., 2008; Bohr et al., 2022), varying with solute size, vascular structure, arousal state, and pathology (Xie et al., 2013; Hladky and Barrand, 2014). Additionally, intramural periarterial drainage (IPAD) along arterial basement membranes provides an alternative and complementary clearance route to the glymphatic model, particularly relevant to cerebral amyloid angiopathy and age-related vascular dysfunction (Weller et al., 1998; Weller et al., 2008; Carare et al., 2008; Keable et al., 2016).

## Subarachnoid compartment, arachnoid barrier, and CSF circulation–recirculation: the mesosegment

The subarachnoid space (SAS) constitutes the dominant extracerebral CSF reservoir and, in a tripartite framework, is an essential mesoscale interface where solutes leaving perivascular microdomains are redistributed, filtered, and routed toward final efflux. However, the CSF-filled SAS has received comparatively little attention as a functionally organized transport compartment, and quantitative *in vivo* data on its regional dynamics and mixing remain limited (Plá et al., 2023; Ringstad and Eide, 2024). Ultra-fast magnetic resonance encephalography (MREG) and dynamic diffusion-weighted MRI now resolve cardiorespiratory-driven pulsatile waves propagating through SAS and perivascular space (Kiviniemi et al., 2016; Wen et al., 2022). Human CSF dynamics is growingly understood as a combination of net production-driven flow (approximately 500 mL/day, with a total volume of approximately 150 mL and 3–4 turnovers daily; Sakka et al., 2011; Xiang et al., 2023) and a prominent pulsatile “to-and-fro” motion that facilitates local mixing and solute exchange rather than a purely unidirectional stream (Brinker et al., 2014). This view is particularly relevant to glymphatic-like clearance, as the SAS acts not merely as a downstream sink but also as a compartment in which CSF-borne macromolecules and immune signals accumulate and are routed toward specific exit pathways (Xiang et al., 2023).

Structurally, the arachnoid is an active mesothelial-/epithelial-like interface that regulates CSF exchange through a junction-sealed barrier and a trabeculated subarachnoid topology that shapes solute mixing and different residence times for the different solutes (Yamada, 2021; Derk et al., 2023; Hartmann et al., 2023). CSF circulation–recirculation reflects oscillatory cardiac–respiratory pulsation-driven flow that sustains interaction with pial, perivascular, and arachnoid structures guiding solute clearance (Milhorat, 1975; Cserr and Patlak, 1992; Brinker et al., 2014; Yamada, 2021).

A major recent advance is the identification of a subarachnoid lymphatic-like membrane (SLYM), a mesothelium-like boundary dividing SAS into superficial and deep compartments and acting as a selective filter and immune-surveillance surface (Møllgård et al., 2023). The SLYM has been proposed to delimit vascular and cisternal territories, modulating the access of CSF-borne solutes to the pia-associated vasculature and deeper SAS channels. This laminar structure provides the morphological substrate for defining a functional “mesosegment,” supporting size- and context-dependent segregation of solutes (including inflammatory mediators and macromolecular waste) prior to reaching terminal efflux pathways (Møllgård et al., 2023; Plá et al., 2023; Figure 1B).

Finally, the SAS should be considered in the context of multiple parallel efflux pathways, which may vary across species and pathological conditions. Experimental tracer studies support that CSF drains through dural lymphatic vessels toward deep cervical lymph nodes, as well as via substantial egress across the cribriform plate into nasal lymphatics (Kida et al., 1993; Koh et al., 2005; Aspelund et al., 2015; Louveau et al., 2015; Ma et al., 2017).

In humans, intrathecal tracer MRI demonstrates trans-arachnoid passage with efflux toward the parasagittal dura along the superior sagittal sinus, indicating a relatively prominent dorsal route that links CSF to the dural meningeal lymphatic network. At the same time, classical venous resorption via arachnoid villi and AGs likely operates in parallel. In contrast to rodents, evidence for substantial *in vivo* CSF outflow along cranial nerves and the cribriform plate in humans is comparatively limited, suggesting that this ventral pathway is less prominent and that human CSF efflux is organized as a multimodal drainage scheme rather than a single dominant route (Milhorat, 1975; Johanson, et al., 2008; Weller et al., 2010; Ringstad and Eide, 2020; Albayram et al., 2022; Ringstad and Eide, 2024). Along the CSF circuit from choroid plexus secretion and periventricular or circumventricular interfaces to perivascular sleeves and the leptomeningeal or subarachnoid compartment, border-associated macrophages in the choroid plexus, perivascular, and meningeal niches provide a distributed layer of innate immune surveillance by sampling CSF-borne cues, phagocytosing debris, and regulating leukocyte trafficking at barrier surfaces, thereby complementing arachnoid and epithelial barrier functions within this arachnoid–CSF interface (Hablitz and Nedergaard, 2021; Silvin et al., 2023; Vara-Pérez and Movahedi, 2025).

## Macrosegment: dural lymphatics and arachnoid granulations as terminal efflux gateways

Tracer studies were pivotal in defining terminal efflux pathways that constitute the macrosegment of the tripartite model. The rediscovery of dural lymphatic vessels (particularly along dural sinuses) has shown that they transport CSF- and ISF-derived macromolecules from the SAS to deep cervical lymph nodes (Aspelund et al., 2015; Louveau et al., 2015). Converging with this, AGs are now viewed as structured egress gateways with immunological connectivity, rather than merely pressure-dependent one-way valves into venous sinuses (Shah et al., 2023). Human AGs harbor immune cells and interface with the dura–arachnoid stroma and adjacent bone marrow, consistent with a lymphatic-associated conduit role at the CSF–dura boundary (Shah et al., 2023). As cited above, classical anatomical tracer studies demonstrated direct CSF outflow through the cribriform plate into nasal lymphatics as a second major lymphatic egress route (Kida et al., 1993; Koh et al., 2005). Collectively, these data underpin the prevailing “glymphatic–lymphatic axis” narrative, reinforcing a key implication for our present review: a substantial fraction of solute elimination is determined by extracerebral CSF handling and route selection, which are likely shaped by subarachnoid compartmentalization and gating (Yasargil, 1984; Johanson et al., 2008; Aspelund et al., 2015). Finally, recent observations in humans suggest that AGs may function as lymphatic-associated conduits interfacing CSF with dura–arachnoid stroma and adjacent marrow spaces, which supports their inclusion within the terminal macrosegment rather than considering them only as venous pressure valves (Shah et al., 2023).

## Discussion

Here, we introduce a micro–meso–macro segmentation that complements the original intraparenchymal–extraparenchymal glymphatic concept (Iliff et al., 2012; Iliff et al., 2015) and the multiscale synthesis linking microscopic determinants (perivascular spaces, astroglial endfeet/AQP4 polarization, and meningeal lymphatic vessels) to macroscopic boundary conditions imposed by the cranial enclosure, the cerebrovascular scaffold, and CSF circulation across brain and spinal compartments (Bohr et al., 2022). The segmentation also remains consistent with more classic barrier-centered models emphasizing CSF–ISF coupling and parallel extracerebral elimination via AGs and cranial/spinal nerves-associated lymphatic routes (Abbott, 2004). Our present human-adapted extension considers the SAS and CSF as primary extracerebral determinants of clearance and access to terminal efflux pathways.

Conceptually, our three-interface model (Figure 1) proposes the micro–meso–macro organization of CSF clearance and immune trafficking, extending the conventional “glymphatic–lymphatic axis” (Hablitz and Nedergaard, 2021; Mestre et al., 2020). It combines *1* the microsegment (“glymphatic”), denoting intraparenchymal perivascular CSF–ISF exchange coupled to astroglial–vascular interfaces; Iliff et al., 2012); *2* the mesosegment (“glymphatic-like”), which designates an intermediate domain formed by extracerebral cisternal and subarachnoid CSF compartments together with perineural and perivascular continuities, in line with classic Cushing´s concept of CSF as a “third circulation” (Cushing, 1925; Milhorat, 1975); *3* the macrosegment (“lymphatic”), which comprises lymphatic efflux routes, including meningeal lymphatic vessels integrated within dural outflow networks operating with AG-mediated CSF egress (Aspelund et al., 2015; Louveau et al., 2015; Proulx, 2021). While the widely used “glymphatic–lymphatic axis” remains a useful heuristic (Hablitz and Nedergaard, 2021; Mestre et al., 2020), it does not fully reconcile with the growing evidence for the multi-compartment, multi-route organization of clearance and immune trafficking described across the micro (perivascular; Figure 1A), meso (subarachnoid/arachnoid; Figure 1B), and macro (dural/AG–lymphatic; Figure 1C) levels within a broader, integrated concept of brain clearance (Kipnis et al., 2025). Reviews of CSF hydrodynamics and outflow anatomy indicate that extracerebral CSF handling is not a passive downstream sink but rather, a key determinant of solute residence time, routing, and final elimination through arachnoid villi/AG dural lymphatics and perineural/nasal lymphatic pathways in parallel (Proulx, 2021). This underpins the central premise of the tripartite model, namely, that effective elimination is an emergent systems property rather than a single-interface phenomenon.

CSF homeostasis appears highly plastic, emerging from distributed production and exchange rather than solely from choroid plexus filtration, with significant extra-choroidal secretion at vascular and periventricular interfaces (Milhorat, 1972; Hladky and Barrand, 2014). Absorption is likewise route-diverse, with the relative roles of AGs, dural lymphatics, and perineural pathways varying across development, physiological states, and diseases (Proulx, 2021). In this plastic framework, the “microsegment” (perivascular exchange) can load solutes into CSF, but onward transport to elimination likely follows multiple anatomical corridors rather than a single perivascular-to-lymphatic continuum (Rasmussen et al., 2022). Integrative reviews of brain fluid transport explicitly describe perivascular exchange as one component within a broader network that includes ventricular/periventricular interfaces (ependymal–periependymal exchange), pial/subpial boundaries, and perineural routes, each with distinct geometry and transport regimes (Milhorat, 1972; Rasmussen et al., 2022).

In our present model, the mesosegment is not confined to a putative mesothelial origin of the arachnoid (Alcolado et al., 1988) but instead designates the subarachnoid compartment as an intermediate pivot interface, encompassing leptomeningeal boundaries (such as SLYM and arachnoid-related interfaces) and cisternal/perivascular geometry, where CSF-borne solutes undergo mixing, compartmentalized partitioning, and immune surveillance before final egress (Møllgård et al., 2023; Eide and Ringstad, 2024). Functionally, this mesoscale behaves as a bidirectional exchange hub coupling the intraparenchymal perivascular network (micro) to macroscale efflux routes, including arachnoid villi/AG draining to dural venous sinuses, dural/meningeal lymphatics, and perineural outflow pathways, where bidirectionality denotes dynamic two-way exchange and regional redistribution within the SAS without implying symmetric net flux (Proulx, 2021; Eide and Ringstad, 2024; Ringstad and Eide, 2024).

Finally, translation from rodents to humans is limited by major anatomical differences in brain size, cortex gyrification, and subarachnoid topology differences, all of which are expected to influence compartmentalization, regional CSF mixing, and residence times (Matsumae et al., 2016; Béchet et al., 2021; Kameya et al., 2024). In rats, the cerebral SAS is largely confined to basal cisterns and periarterial channels, with intracranial CSF volumes of approximately 0.2–0.3 mL, whereas in humans, MRI-based volumetry estimates approximately 250 mL intracranial CSF plus approximately 85 mL spinal CSF (total approximately 335 mL), of which approximately 30 mL resides in ventricles and 110 mL in SAS, turning over 1.5–1.8 times per day at production rates of 0.35–0.4 mL/min (Sakka et al., 2011; Xiang et al., 2023). Therefore, a simple, tightly coupled glymphatic–lymphatic axis may overfit rodent tracer data while underestimating both the mesosegment’s role (SAS compartmentalization/gating) and the macrosegment’s diversity (AG–dural–perineural lymphatic routes) in the human CNS.

## Conclusion

The emergence of glymphatic concepts has added a new functional dimension to the SAS, indicating that its traditional portrayal as primarily protective and cushioning is no longer sufficient as a standalone description. In humans, CSF (and the magnitude and spatiotemporal organization of its volumetric flow) should be regarded as a primary determinant of solute and toxic-protein clearance within the glymphatic–lymphatic continuum (Rasmussen et al., 2022). The SAS acts as a pivotal intermediate compartment (Iliff et al., 2015) in which CSF recirculation, SAS compartmentalization, and macroscopic outflow routing shape CSF redistribution, solute residence time, and effective access to terminal egress pathways. Simplified parenchyma-to-lymphatic models fail to capture these controlling steps, motivating the development of explicitly segmented conceptual frameworks (Iliif et al., 2012; Proulx, 2021). The proposed three-interface conceptual model operationalizes this systems-level complexity by treating clearance as a sequence of partially dissociable control points across parenchymal, subarachnoid, and dural outflow interfaces, forming a global brain-clearance system.

Clinically, this organization supports therapeutic stratification and nosological precision because different disorders may preferentially disrupt different interfaces: glymphatic dysfunction at the micro (perivascular–astroglial) interface is increasingly linked to impaired parenchymal waste handling in cerebral small-vessel disease and neurodegenerative proteinopathies, whereas iNPH/hydrocephalus phenotypes map more naturally onto mesoscale CSF–SAS transport and extracerebral tracer handling (Mestre et al., 2017; Patel et al., 2019; Proulx, 2021), as suggested by intrathecal gadolinium MRI studies that identify major bottlenecks at parasagittal dura and lymphatic outflow pathways in humans (Vinje et al., 2023). Moreover, the model predicts a macro-to-micro coupling whereby slowed or obstructed dural/cervical lymphatic outflow imposes upstream “back-pressure” on extracerebral efflux, secondarily dampening perivascular clearance and promoting retrograde accumulation of soluble toxic proteins. Together with evidence for multiple parallel CSF outflow routes and human meningeal lymphatic–extracranial connections, the model implies clinically exploitable pathway redundancy (“clearance plasticity”) under stress (Mestre et al., 2017; Proulx, 2021; Albayram et al., 2022). Further progress will require integrative, quantitative multiscale validation using multi-compartment mathematical models and poroelastic or dispersion-aware frameworks of CSF–ISF transport that already show how surface membranes and efflux boundaries can act as central bottlenecks for clearance (Martinac and Bilston, 2020; Ray et al., 2021; Bork et al., 2024; Guo et al., 2025). Subject-specific CSF flow simulations in cranial and spinal SAS, constrained by *in vivo* human imaging, will be needed to identify segment-specific bottlenecks and predict intervention effects (Gupta et al., 2010; Bohr et al., 2022).
